# VWCE Functions as a Tumor Suppressor in Breast Cancer Cells

**DOI:** 10.3389/fonc.2020.586342

**Published:** 2020-10-22

**Authors:** Dan Zhang, Lili Wan, Fan Yang, Wenlan Liu, Litao Liu, Shengnan He, Ni Xie

**Affiliations:** Health Science Center, Biobank Shenzhen Second People’s Hospital, The First Affiliated Hospital of Shenzhen University, Shenzhen, China

**Keywords:** breast cancer cells, Von Willebrand factor C and EGF domain, tumor suppressor, WD-repeat domain 1, metastasis

## Abstract

Breast cancer remains a leading cause of cancer-related death, for which the majority of deaths result from metastases. Von Willebrand factor C and EGF domain (VWCE) is a member of the Von Willebrand factor (VWF) gene family; however, its function, regulatory mechanism, and clinical value in breast cancer remain unclear. In the present study, we sought to elucidate the role of VWCE in breast cancer metastasis. We examined the expression of VWCE in breast cancer tissues and normal control tissues of 50 breast cancer patients. We found that VWCE expression was downregulated in breast cancer cells and tissues compared to normal breast epithelial cells or the adjacent normal tissues. To explore the role of VWCE in human breast cancer development, we introduced a VWCE-overexpressing or control lentiviral vector into the breast cancer MDA-MB-453 and MDA-MB-231 lines *in vitro*. The overexpression of VWCE inhibited the proliferation, migration, invasion, and chemoresistance of the breast cancer cell lines. More importantly, the forced expression of VWCE suppressed tumor formation and metastasis in nude mice. iTRAQ-based quantitative proteomic analysis revealed that VWCE overexpression induced a 10-fold decrease in the level of WD-repeat domain 1 (WDR1) protein expression. Rescue experiments further verified that WDR1 was a downstream molecule of VWCE, and WDR1 overexpression reversed the above effects of VWCE overexpression on tumor growth. Therefore, VWCE may represent a novel tumor suppressor, for which its deregulation promotes breast cancer progression *via* the upregulation of WDR1.

## Introduction

Breast cancer is the most common type of cancer affecting women worldwide and the leading cause of cancer-related death in women (1). Moreover, metastasis is the leading cause of death in breast cancer patients, accounting for 90% of breast cancer mortality (2). Although significant progress has been made in breast cancer research over the past decade, our understanding of metastasis remains limited and it is not yet possible to prevent and provide targeted treatment for metastases (3). Therefore, there is an urgent need to elucidate the potential molecular pathways and associated mechanisms that contribute to the progression of breast cancer metastasis.

VWCE is a member of the Von Willebrand factor (VWF) gene family (4), also called URG11. Previous studies have demonstrated that VWF gene polymorphisms play an important role in a variety of physiological and pathological processes, including embryonic development, angiogenesis, physiological hemostasis, genetic hereditary diseases, and malignant tumors (5–12). In addition, the role of VWCE has been studied in several types of tumors. For example, VWCE has been found to be highly expressed in hepatocellular carcinoma, gastric cancer, pancreatic cancer, and lung cancer, and is associated with increased invasion and metastasis of these tumor cells (13–18). It has also been found that the N-terminal VWC domain of VWCE binds to bone morphogenetic proteins (BMPs) with a high affinity (10), which have been linked to the transformation of breast cancer subtypes (11). Notably, BMP signal transduction mediated by a new peptide agonist, P123, leads to the reversal of the epithelial-mesenchymal transition process in human breast cancer stem cells and inhibits self-renewal and growth, thereby slowing the invasion and metastasis of breast cancer (12). However, to date, there have been no reports on the role of VWCE in breast cancer. In this study, the expression and function of VWCE in breast cancer cells are studied, and the possible anti-tumor mechanisms are further explored.

## Materials and Methods

### Patient Tissue Specimens

This study was approved by the clinical research medical ethics committee of the First Affiliated Hospital of Shenzhen University. After obtaining written informed consent, breast cancer tissues and matched adjacent non-cancerous tissues were collected from 50 patients at the First Affiliated Hospital of Shenzhen University (The Second People’s Hospital of Shenzhen, Shenzhen 518037, China). All tumor specimens were obtained from new patients who were not treated prior to surgery (study period: 2017–2018); Tissues were fixed in RNAlater and stored at -80°C until further use (19). Pathology reports were provided by the Shenzhen Second People’s Hospital Cancer Center.

### Cell Lines

Five breast cancer cell lines with different hormone receptor profiles were used in this study: 1)MCF7; 2) BT-474; 3) SK-BR-3; 4) MDA-MB-231; and 5) MDA-MB-453. In addition, MCF-10A cells are normal breast epithelial cells, used as a control. All cell lines were purchased from the Chinese Science Shanghai Institute of Cell Biology and cultured in L-15/1640/Dulbecco’s modified Eagle medium (DMEM; Gibco, Carlsbad, CA, USA) supplemented with 10% fetal bovine serum (Gibco) at 37°C in a humidified atmosphere with 5% CO2.

### Quantitative Real-Time PCR

Quantitative real-time RT-PCR (qRT-PCR) was performed using SYBR Premix Ex Taq™п (TaKaRa), and the expression of β-actin was used as an internal control. The assay was conducted in triplicate using the following primer sets: VWCE forward, 5′-ACG GAA ATG TGG CAT TCA GCAAAG-3′, VWCE reverse, 5′-CGGGCTTGTAGGTAAAGTCTGTGT-3′, product size 179 bp; β-actin (used as the internal primer) forward, 5′-GATCATTGC TCCTCCTGAGC-3′, reverse, 5′-ACTCCTGCTTGCTGATCCAC-3′, product size 101 bp. Amplification was carried out in 20 μl reaction systems, comprised of SYBR Green Mix (16.4 μl), cDNA (2 μl), and 0.8 μl of the forward and reverse primer (10 μM) under the following conditions: 95°C pre-denaturation for 30 s, followed by 40 cycles of denaturation at 95°C for 5 s, and annealing/extension at 60°C for 34 s. All reactions were conducted in duplicate. The relative expression of VWCE was calculated using the 2−ΔΔCT method.

### Western Blot

We randomly selected six pairs of tissues that were lysed along with the six breast cell lines using 500 μl of the cell lysate (100 μl containing RIPA buffer with 1 μl phenylmethylsulfonyl fluoride). The total protein concentration was determined by the BCA method, and 20 μg of each protein sample was separated by 10% sodium dodecyl sulfate-polyacrylamide gel electrophoresis. Proteins were transferred to polyvinylidene fluoride membranes using the semi-dry method. The membranes were blocked with 5% bovine serum albumin, treated with a rabbit anti-human VWCE polyclonal antibody (1:500 dilution) (Sigma Aldrich, St. Louis, MO, USA) and, GAPDH polyclonal antibody (1:5,000 dilution) (CST, USA) and β-tublin polyclonal antibody (1:5,000 dilution) (Abcam, Cambridge, UK) overnight at 4°C, washed three times with TBST for 10 min each, and treated with horse radish peroxidase (HRP)-labeled goat anti-Rabbit IgG at room temperature for 1 h. The immunocomplexes were then visualized by chemiluminescent imaging.

### Immunohistochemical Analysis

A total of 87 paraffin-embedded breast cancer tissues and the corresponding adjacent tissue microarray were purchased from Alenabio (Xi’an, China). Briefly, after deparaffinization and rehydration, tissue slides were subjected to antigen retrieval by boiling in citrate buffer (10 mM, pH 6.0) and immersed in 30% hydroxyl peroxide (H2O2) to quench the endogenous peroxidase activity. After washing in phosphate-buffered saline, the slides were incubated with an anti-VWCE antibody (1:500, Sigma-Aldrich) overnight at 4°C, followed by an HRP-conjugated goat anti-rabbit IgG antibody (sc-2030; Santa Cruz Biotechnology, Dallas, TX, USA) at room temperature. The samples were then stained with a freshly prepared DAB solution (Dako Pure Chemicals, Glostrup, Denmark) and counterstained with hematoxylin. All of the stained sections were assessed for the degree of immunostaining and scored by two pathologists. Five fields of vision were randomly selected under the microscope. The presence of brown staining in the cytosol was considered to be positive for VWCE expression, regardless of the intensity. The sections were scored as follows: 0 points, 0 positively stained cells; 1 point, < 25% of the cells were stained positive; 2 points, 25%−50% of the cells were stained positive; 3 points, 50%−75% of the cells were stained positive; and 4 points, > 75% of the cells were stained positive. Moreover, the staining intensity was divided into four groups: 0, negative; 1, weak positive; 2, moderate positive; and 3, strong positive. The score for each tissue was the product of the two integrals. The results were determined as follows: I (0), II (1 – 4), III (5 – 8), and IV (9 – 12), where I was considered to be negative and II, III, and IV were considered to be positively stained.

### Cell Transfection

Cells were plated approximately 24 h prior to transfection to achieve 80%–90% confluence at the time of transfection. Each cell line was transfected with VWCE and WDR1-overexpressing lentiviral vectors and a corresponding negative control virus, respectively, using transfection reagent (GENE, Shanghai, China) according to the manufacturer’s instructions.

### CCK8 Assay

The cell suspensions were adjusted to 2,000 cells/ml and seeded into 96-well plates at 100 μl per well. After an incubation for 6−8 h to ensure cell adherence, the cell growth rate was determined using Cell Counting Kit-8 (CCK-8; CCK8: medium, 1:10). After an incubation at 37°C for 2 h, the light absorbance at 450 nm was measured for each sample. The OD (450) value was taken as the absorbance value at Day 0. Cell absorbance values were recorded on days 1, 2, 3, and 4.

### Colony Formation Assay

Cell suspensions were adjusted to 1,500 cells/ul and seeded into six-well plates at 1 μl/well. After cultivation for 7−10 days, the medium was removed, and the cells were fixed with 4% paraformaldehyde (1 ml) at 37°C for 15 min, and then cells were stained with 0.1% crystal violet at room temperature for 20 min and washed with water 2−3 times prior to imaging (20).

### Transwell Migration Assay

To assess the level of cellular migration, cells in the logarithmic growth phase were routinely harvested by digestion and centrifugation. The cells were resuspended in serum-free medium, and adjusted to 1 × 10^5^/ml. Then, 200 μl of each suspension was added to the upper Transwell chamber, whereas 500 μl medium containing serum was added to the lower chamber. After 24 h, the non-migrated cells on the upper surface of the filters were wiped off using a cotton swab. Migratory cells on the lower membrane surface were fixed with 4% paraformaldehyde for 15 min, stained with 1% crystal violet for 20 min, washed with single distilled water, and dried. Cells were then visualized with an OLYMPUS IX71 Inverted Microscope and the number of migratory cells was counted. Data were presented as the means ± standard deviations of the results from three independent experiments.

### Wound-Healing Assay

The cells were planted in a six-well plate at a certain density, and the cells were incubated in a medium containing 0.5% FBS at 37°C (21). They reached a monolayer confluence of about 80%, using a 10 μl pipette. The suction tip scrapes the cell monolayer to prepare the wound. After the wound appears, wash twice with PBS to remove the separated cells and replace it with fresh serum-free medium. The wound area is photographed under an inverted microscope within a specified time. Use image software to quantify and analyze the width of the gap. The average wound size represents the level of relative cell migration (22).

### Drug Resistance Clonogenic Assay

The cells were treated with a single dose of docetaxel for one week. The resistant clones were fixed in 4% paraformaldehyde and stained with 0.1% crystal violet and counted.

### Cell Cycle Analysis

After the cells grow to 70%–80%, digest and centrifuge to collect the cells, add pre-cooled ethanol to fix. The cells were washed with pre-chilled PBS and resuspended with 500 μl of PBS containing staining buffer, 10 ul RNase A and 25 ul propidium iodide. The cell suspension was evaluated by flow cytometry.

### iTRAQ-Based Proteomics Analysis

iTRAQ-based proteomics analyses were performed in MDA-MB-231 cells. Briefly, the cells were dissolved in lysis buffer and labeled with iTRAQ-labeling reagents. After 2D LC and tandem mass spectrometry analyses, protein identification and relative iTRAQ quantification were performed with ProteinPilot™ Software 4.2 (AB SCIEX) using the Paragon™ algorithm for peptide identification. Results with iTRAQ ratio cut-off values of 1.2 and 0.8 for the fold-change and number of cut-off values of three quantifiable peptides for in-protein abundance were accepted.

### Construction of a Breast Cancer Xenograft

BALB/c-nude mice (4–5 weeks of age; 18–20 g) were purchased from the Center of Experimental Animals of Guangdong province, China, and housed in a sterile environment. For the tumor formation experiment, a total of 16 four-week-old female BALB/c-nude mice were randomly divided into two groups (n = 8/group): 1) MDA-MB-231 cells (8 × 10^6^) with the stable expression of VWCE or a control vector were subcutaneously injected into the right flanks of the nude mice, respectively. The tumor volume was determined using the eq. V = 0.5 × D × d2 (V, volume; D, longitudinal diameter; d, latitudinal diameter). The developing tumors were observed over the next 33 days.

### Construction of a Xenograft Metastasis Model

MDA-MB-231 cells stably expressing luciferase (referred to as MDA-MB-231-luc) were transfected to express VWCE. In the lung metastasis experiment, with a density of 1 × 10^6^ MDA-MB-231 cells expressing luciferase ovexpressing VWCE were suspended in 100 μl PBS and injected into the tail vein of six-week-old BALB/c nude mice. Six weeks later, IVIS-200 camera system was used to analyze the metastatic disease of mice by bioluminescence imaging to detect the expression of luciferase, the nude mice were dissected, and lung tissues were collected. For the histological examination, pulmonary tissues were fixed in 10% neutral-buffered formalin and embedded in paraffin. Then, four resections were prepared for H&E staining and an immunohistological assay. E-cadherin was detected using an anti-E-cadherin antibody. All experimental animal procedures were approved by the Institutional Animal Care and Use Committee of The Second People’s Hospital of ShenZhen, China.

### Database Analysis

The BRCA datasets and expression of the target genes VWCE by mRNA sequencing were downloaded from The Cancer Genome Atlas (TCGA) dataset. The expression data between breast cancer tissues and adjacent normal breast tissues were compared using the edgeR package in R. Use the website (http://www.kmplot.com) to identify the prognosis of VWCE in breast cancer patients.

### Statistical Analysis

Data were presented as the means of the results from at least three independent experiments. Correlations between the level of VWCE expression and various clinicopathological parameters were analyzed using a χ2 test. Differences between VWCE-overexpression and the negative control of the lentivirus-transfected cells in the CCK-8 assay were analyzed by a one-way analysis of variance (ANOVA). All other data were analyzed using independent sample *t-*tests. All statistical analyses were performed using the SPSS 21.0 software package (SPSS Statistics Inc., Chicago, IL, USA), and a threshold of p < 0.05 was defined as significant for all tests.

## Results

### VWCE Is Down-Regulated in Breast Cancer Tissues and Cells

**Figure 1:**
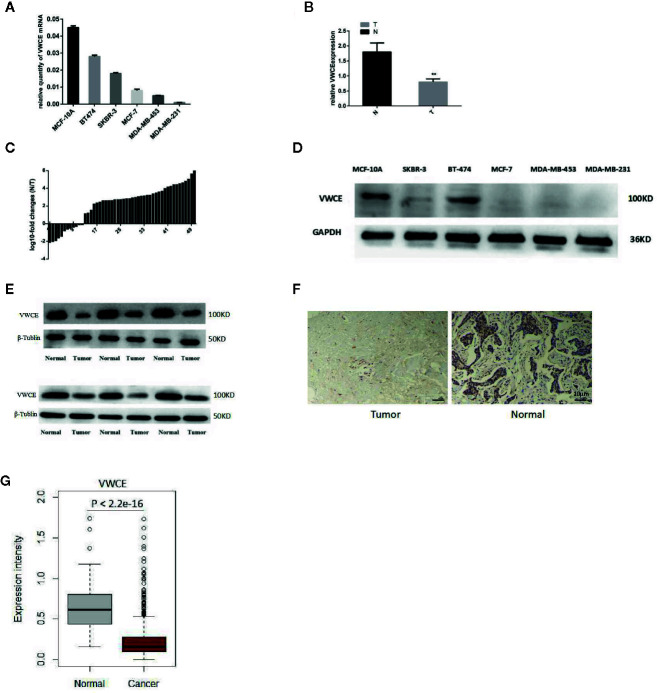
Down-regulation of Von Willebrand factor C and EGF domain (VWCE) in breast cancer tissues and breast cancer cell lines. **(A)** Quantitative real-time RT (RT-PCR) analysis of VWCE expression in five breast cancer cell lines (MCF-7, BT474, SKBR-3, MDA-MB-231, and MDA-MB-453) and a normal breast cell line (MCF-10A). **(B)** qRT-PCR analysis of VWCE mRNA expression in breast cancer tissues and matched normal control tissues. T, tumor; N, normal. **(C)** The expression of VWCE was analyzed in samples from 50 breast cancer patients. The height of the columns in the chart represents log_10_-transformed fold-changes (normal and cancer tissue) in VWCE expression. **(D)** Western blot analysis of VWCE protein expression in the five breast cancer cell lines and in MCF-10A cells. **(E)** Western blot analysis of VWCE protein expression in fresh breast cancer and adjacent non-tumor tissues. **(F)** Representative immunohistochemical staining of VWCE protein expression in breast cancer (right) and matched non-tumor tissues (left) (cale bar = 10 μM). **(G)** The level of VWCE mRNA expression was compared between the tumor and normal group of TCGA breast cancer. **P < 0.01.

We first analyzed the level of VWCE expression in breast cancer tissues and cultured the breast cancer cell lines using qRT-PCR, immunohistochemistry, and Western blot. The level of VWCE mRNA expression was lower in moderately invasive cells (MCF-7 and BT474) than in non-tumorigenic MCF-10A cells, and was the lowest in highly invasive cancer cell lines (MDA-MB-453, SKBR-3, and MDA-MB-231), as assessed by reverse transcription-PCR (**Figure 1A**). Similarly, using qRT-PCR, we assessed the level of VWCE mRNA expression in 50 human breast cancer specimens relative to 50 normal breast tissue samples, and VWCE mRNA is highly expressed in normal tissues adjacent to the tumor (**Figure 1B**); However, in contrast, the expression in breast cancer tissue is lower. The level of VWCE mRNA expression in breast cancer tissues and matched adjacent non-cancerous tissues (n = 50) was assessed by qRT-PCR analysis. In most patients, there was a positive fold-change in the level of VWCE expression (**Figure 1C**).

We next examined the level of VWCE protein expression in breast cancer cell lines (**Figure 1D**) and tumor tissues (**Figure 1E**), which was consistent with our VWCE mRNA expression data. In order to examine the level of VWCE protein expression in human breast cancer, we performed an immunohistochemical analysis of the purchased tissue microarray, which consisted of 87 breast cancer tissues and 87matched normal breast tissue samples. Staining was performed using VWCE specific antibodies, and at least two pathologists scored the staining intensity. Quantification of the mean of the tumor-adjacent normal breast tissues exhibited a significantly higher level of VWCE expression than that of the breast cancer tissues (**Figure 1F**). These data are consistent with our results regarding the level of VWCE mRNA expression. The positive expression rate of VWCE among the tested breast cancer and adjacent normal tissues was 39.08% (34/87) and 59.77% (52/87), respectively.

We also used the TCGA datasets to examine the level of VWCE mRNA expression in 1,079 human breast cancer specimens relative to the 113 normal breast tissues (**Figure 1G**). Data from the same tissue sample but from different vials was averaged. The gene expression data was generated *via* RNAseq. We further confirmed that VWCE exhibited lower levels of expression in breast cancer tissues compared to the adjacent normal tissues in breast cancer patients.

### VWCE Overexpression Inhibits the Proliferation of Breast Cancer Cells

**Figure 2:**
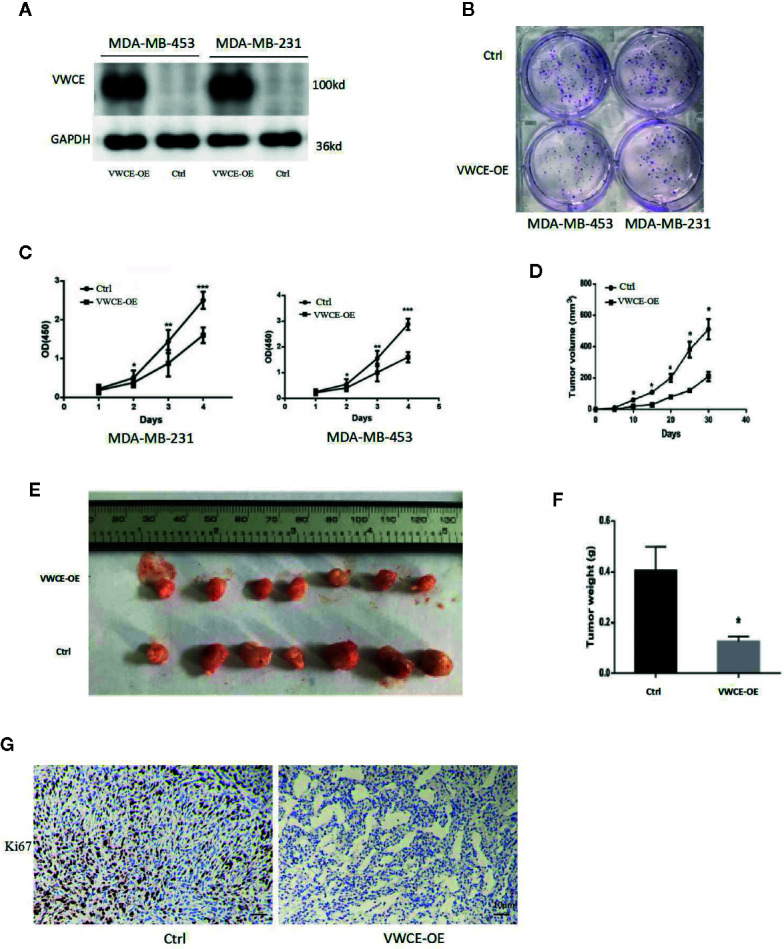
Von Willebrand factor C and EGF domain (VWCE) inhibits breast cancer cell proliferation. **(A)** Western blot analysis of VWCE protein expression in MDA-MB-453 and MDA-MB-231 cells stably transfected with a VWCE-overexpressing or negative control lentivirus. GAPDH was used as a loading control. **(B)** A plate cell colony formation assay of the ectopic expression of VWCE on MDA-MB-453 and MDA-MB-231 cells. **(C)** CCK8 analysis of the effects of the ectopic expression of VWCE on the proliferation of MDA-MB-453 and MDA-MB-231 cells. Experiments were performed in triplicate and data are presented as means ± standard deviations; *p < 0.05, **p < 0.01, and ***p < 0.001, compared to the control, respectively. **(D)** The tumor growth curves were measured after a subcutaneous injection of the MDA-MB-231-Control and MDA-MB-231-VWCE. The tumor volume was calculated every five days. Error bars indicate standard deviation (Student’s t-test; *P < 0.05; n = 5). **(E)** Photographs of the dissected tumors from nude mice. **(F)** The weight of tumors from mice with MDA-MB-231-Control and MDA-MB-231-VWCE implantation. Error bars indicate standard deviation (Student’s *t*-test; *P < 0.05, n = 5). **(G)** Cellular proliferation in VWCE-expressing vs. control tumors. Paraffin-embedded tissue sections of the primary tumors from mice injected with MDA-MB-231-Control and MDA-MB-231-VWCE cells were immunostained with an anti-Ki67 antibody. Photomicrographs were taken at 20× magnification; scale bars = 10 μM.

We next transfected MDA-MB-453 and MDA-MB-231 cells with a VWCE-overexpressing lentivirus and control virus to analyze the role of VWCEof proliferation in breast cancer cells. The efficacy of transfection was examined by a Western blot analysis (**Figure 2A**). As expected, the level of VWCE protein expression in the cells of the experimental groups exhibited significantly higher levels of VWCE expression compared to those in the negative control groups. VWCE overexpression significantly inhibited the proliferation of MDA-MB-453 and MDA-MB-231 cells compared with the negative control groups (p < 0.05), as determined by a cancer cell colony formation assay (**Figure 2B**). Moreover, the CCK-8 assay showed that the ectopic expression of VWCE significantly inhibited MDA-MB-453, and MDA-MB-231 cell proliferation in a time-dependent manner, compared with the negative control transfection groups (p < 0.001) (**Figure 2C**). In addition, to further assess the effect of VWCE overexpression on tumorigenicity *in vivo*, we subcutaneously injected MDA-MB-231-GFP and MDA-MB-231-VWCE cells into nude mice. Intriguingly, as shown in **Figures 2D, E**, the tumors formed by MDA-MB-231-VWCE cells were significantly smaller than that of the control cells. The average tumor weight of the VWCE-transfected cells in the inoculated mice was significantly decreased compared with that of the control cells on Day 30 (**Figure 2F**). These results suggest that VWCE inhibits tumor growth. To investigate whether cells expressing VWCE are less proliferating *in vivo*, we took immunohistochemical staining of Ki67 (a marker of cell proliferation) from tumors of nude mice. Tumors expressing VWCE showed Ki67 staining significantly lower than controls expressing GFP (**Figure 2G**). These indicating that VWCE may acts as a tumor suppressor *in vivo*, supporting a role for VWCE in the regulation of tumor growth *via* its effects on cellular proliferation.

### VWCE Overexpression Inhibits the Cellular Migration of Breast Cancer Cells

**Figure 3:**
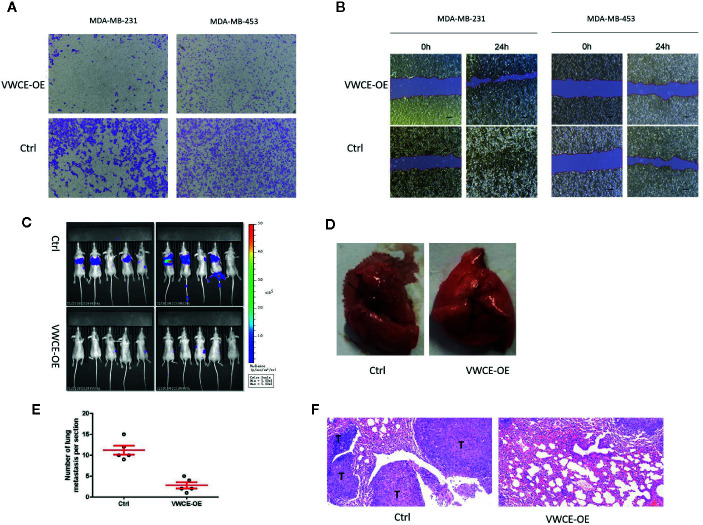
Von Willebrand factor C and EGF domain (VWCE) inhibited the cell migration of breast cancer cells. **(A)** The invasion ability of MDA-MB-231 and MDA-MB-453 cells infected with either the VWCE-overexpression or vector control lentivirus detected by a transwell assay (scale bar = 100 μM). **(B)** The impact of stably up-regulated VWCE expression on cellular invasion in MDA-MB-231 and MDA-MB-453 breast cancer cell lines by a wound healing assay. **(C)** *In vivo* imaging of the control and the VWCE-OE group in the metastatic model was created by tail vein injection. **(D)** The black arrows indicate lung metastatic lesions. **(E)** lung metastases were counted, quantification of lung metastasis in VWCE-overexpression mice compared to contorl mice. **(F)** Lung tissues were photographed, ﬁxed, and stained with hematoxylin and eosin (H&E); scale bar: 100 μm.

To investigate the function of VWCE in breast cancer, we first transfected the MDA-MB-231 and MDA-MB-453 cell lines using a control and VWCE expression vector, and verified VWCE expression by Western blot and qRT-PCR. We then investigated the effect of VWCE on breast cancer cell metastasis. Transwell assays with or without matrix gel showed that VWCE suppressed the migration and invasion ability of MDA-MB-231 and MDA-MB-453 cells (**Figure 3A**). Moreover, while the overexpression of VWCE robustly accelerated wound closure in all of the analyzed breast cancer cell lines, cell motility was also effected (**Figure 3B**). VWCE overexpression also reduced the number of metastatic nodules when MDA-MB-231 cells were injected into the tail veins of mice (**Figure 3C**). To further confirm the effect of VWCE on breast cancer metastasis, we injected MDA-MB-231 breast cancer cells overexpressed with VWCE into the nude mice through the tail vein. The histological analysis of the lung tissue of nude mice was carried out. Lung tissue was extracted and sectioned (**Figures 3D, E**). H&E staining showed that the lungs of mice injected with VWCE overexpressing breast cancer cells had almost no metastasis (**Figure 3F**). In contrast, the lungs of mice injected with vehicle-controlled breast cancer cells were severely infiltrated by metastatic foci. Further pathological examination of lung metastases in mice carrying MDA-MB-231 xenografts overexpressing VWCE showed that the larger ones were observed in nude mice with control MDA-MB-231-luc xenografts compared with local invasive metastases, these metastases are smaller and separate. These results suggest that VWCE can inhibit the metastasis of breast cancer cells *in vivo* and is an important regulator of breast cancer cell invasion and metastasis *in vivo*.

### VWCE Overexpression Induces the Reversal of EMT to MET in Aggressive Breast Cancer Cells

**Figure 4:**
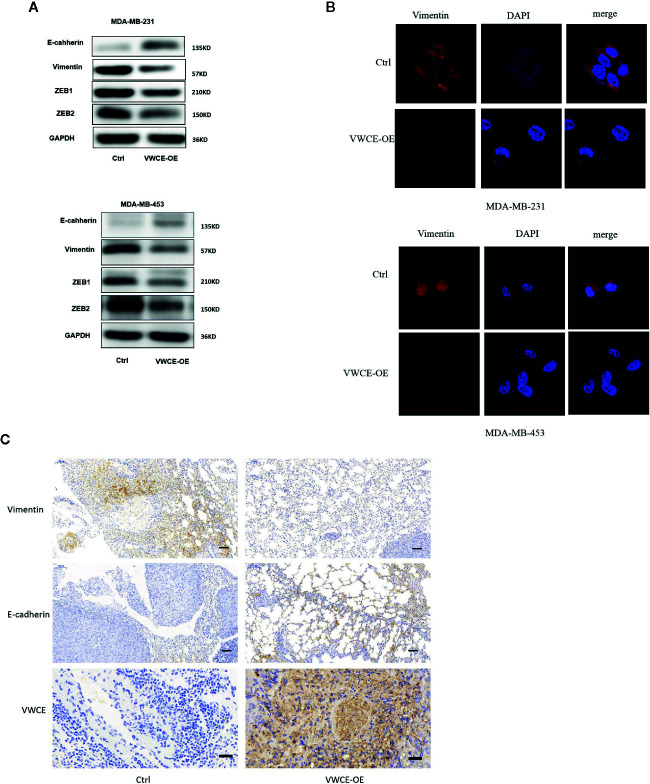
Von Willebrand factor C and EGF domain (VWCE) causes the reversal of EMT to MET in aggressive breast cancer cells. **(A)** EMT molecule markers were determined by Western blot. **(B)** Vimentin expression were evaluated by immunofluorescence in MDA-MB-231 and MDA-MB-453 cells treated by VWCE-overexpression or a control vector.(scale bar =100 μM) **(C)** Representative E-cadherin, vimentin, and VWCE expression in the lung metastases of a nude mouse model by immunohistochemistry (scale bar =100 μM).

We next examined the expression of EMT markers, a characteristic used to define the aggressiveness of breast cancer cells. We selected two breast cancer cell lines that represent the mesenchymal phenotype, MDA-MB-231 and MDA-MB-435 cells, to elucidate the mechanism by which VWCE mediates its anticancer effects. We found that VWCE-overexpression resulted in a significant downregulation of the mesenchymal markers, vimentin, ZEB1, and ZEB2 in both MDA-MB-231 and MDA-MB-453 cells with the concomitant highly significant upregulation of the epithelial marker, E-cadherin, in both the cell lines (**Figures 4A, B**). These results suggest an effective switch from the MET phenotype of breast cancer cells following VWCE overexpression. We also conducted an immunohistochemical analysis to study the *in vivo* effects of VWCE on MET in our xenograft model (**Figure 4C**). Immunostaining for EMT markers confirmed the reversal of EMT by VWCE, in which VWCE-overexpression in the treated tumors had upregulated E-cadherin and decreased vimentin. These results suggest that VWCE overexpression reverses the EMT that occurs during lung metastasis of breast cancer *in vivo*.

### VWCE Overexpression Causes Cell Cycle Arrest at the G1 Phase and Inhibits Chemoresistance in Breast Cancer Cells

**Figure 5:**
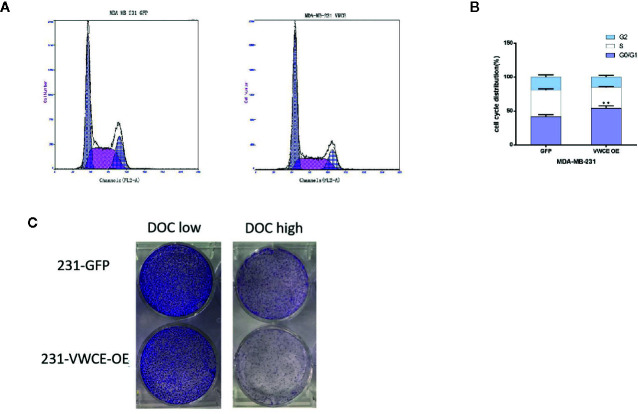
Von Willebrand factor C and EGF domain (VWCE) causes cell cycle arrest at the G1 phase and inhibits chemoresistance in breast cancer cells. Overexpression of VWCE inhibits cell cycle progression in MD-MBA-231 cells. **(A**, **B)** Flow-cytometry analysis showed that the overexpression of VWCE significantly causes a G0/G1 phase cell cycle arrest in MD-MBA-231 cells.**P < 0.01. **(C)** Clone formation in the control or over-expressing VWCE cells following treatment with DOC (2.5 nM) and DOC (5 nM) for five days.

We used flow cytometry to evaluate whether VWCE regulates cell cycle progression in MDA-MB-231 cells treated with a control or VWCE-expressing vector. VWCE induced cell cycle arrest at the G0/G1 phase in MDA-MB-231 cells after 48 h of treatment (**Figure 5A**). The percentage of cells in the G0/G1 phase in the control and VWCE-expressing groups was 41%, and 54%, respectively (**Figure 5B**; P < 0.01). Overcoming resistance to chemotherapy is one of the fundamental issues affecting clinical treatment. Therefore, we examined the effect of VWCE on chemoresistance. We selected Docetaxel (DOC, taxus chemotherapy drug) to assess the chemoresistance of breast cancer cell lines by a colony formation assay. Compared with the control cells, the VWCE-OE cells exhibited lower cell viability under DOC treatment. In the colony formation assays, VWCE-OE cells exhibited a reduced capacity compared to the control cells to form colonies upon DOC treatment (**Figure 5C**). Finally, these results showed that the overexpression of VWCE inhibits chemoresistance.

### Overexpression of WDR1 Partially Reverses the Tumor-Suppressor Role of VWCE in Breast Cancer Cells

**Figure 6:**
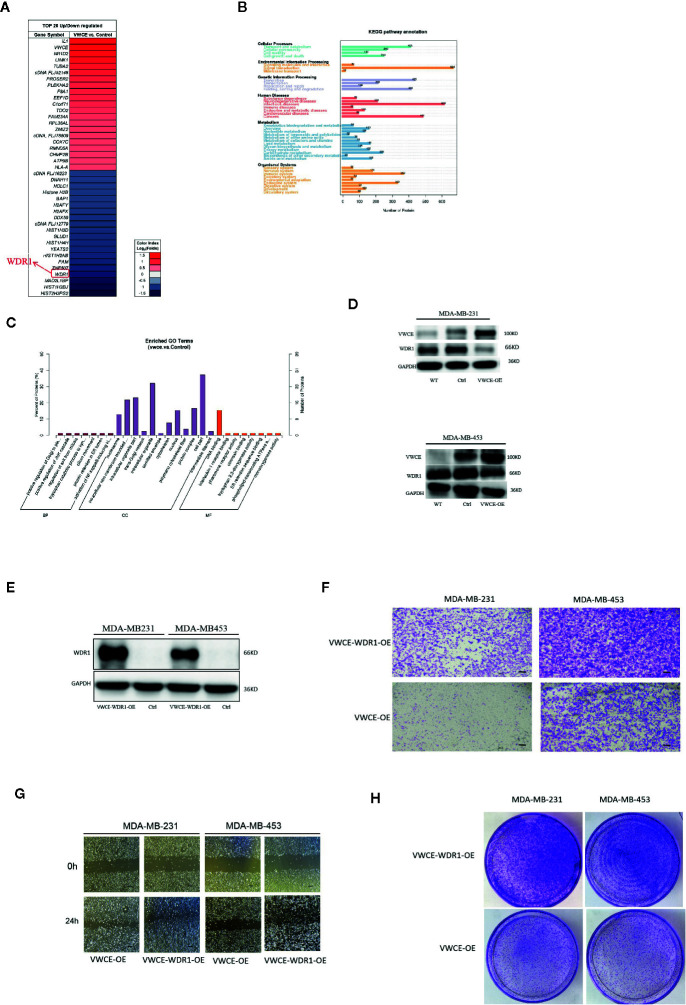
Overexpression of WDR1 partially reversed the tumor-suppressor role of Von Willebrand factor C and EGF domain (VWCE) in breast cancer cells. Identification of differentially-expressed proteins (DEPs) between VWCE overexpression and control groups using an iTRAQ-based proteomic analysis. **(A)** A heat map showing the DEPs between VWCE overexpression (231-VWCE) and control (231-Contorl) groups of breast cancer cell lines. Blue represents lower levels of protein expression and red represents higher levels of expression compared to the control. **(B)** A KEGG pathway analysis of the DEPs. **(C)** A GO analysis of the DEPs. **(D)** Relative expression of VWCE and WDR1 in MDA-MB-231 and MDA-MB-453 cell lines by Western blot. **(E)** Western blot analysis of WDR1 protein expression in MDA-MB-453-VWCE, and MDA-MB-231-VWCE cells were stably transfected with the WDR1-overexpressing or negative control lentiviruses. GAPDH was used as a loading control. **(F)** The impact of WDR1 overexpressionon cell invasion in MDA-MB-453 and MDA-MB-231 cell lines using a transwell assay(scale bar =100 μM). **(G)** The impact of WDR1 overexpression on cell migration in MDA-MB-453 and MDA-MB-231 cell lines using a wound healing assay (scale bar =100 μM). **(H)** The impact of WDR1 overexpression on cellular proliferation in MDA-MB-453 and MDA-MB-231 cell lines using a plate cell colony formation assay.

In order to explore the possible mechanism of the function of VWCE in breast cancer, the total protein of two groups (MDA-MB-231-Control and MDA-MB-231-VWCE cells) was extracted for iTRAQ analysis to identify the VWCE of breast cancer cell lines The differentially expressed protein (DEP) between the overexpression group and the control group. A total of 6163 proteins were detected using iTRAQ. Compared with the NC group, the expression levels of 101 proteins in the VWCE group were significantly different. The layered clustering heat map of differentially expressed proteins is shown in **Figure 6A**. The David database was used to classify the DEPs’ identified biological processes (BP), cellular components (CC), and molecular functions (MF) (**Figure 6B**). In the BP category, protein phosphorylation/oxidation-reduction process/signal transduction/metabolic processes were over-represented. In the CC category, the nucleus/integral component of the membrane/membrane/intracellular were highlighted. In the MF category, protein binding/ATP binding/nucleic acid binding/zinc ion binding were over-represented. In the analysis of the KEGG pathway, the top five pathways included signal transduction, infectious diseases, cancers, translation, as well as folding, sorting, and degradation, in which WDR1 was the prior participant without exception (**Figure 6C**). Consequently, based on the combined results of proteomics, GO, and KEGG pathway analyses, we selected WDR1 as the downstream target of VWCE for further validation with *in vitro* assays. To explore how VWCE and WDR1 inﬂuenced each other in breast cancer cells, a Western blot was performed. The results showed that the overexpression of VWCE decreased the level of WDR1 protein expression (**Figure 6D**). A WDR1-overexpressing lentivirus and the control virus were then transfected into VWCE-MDA-MB-453 and VWCE-MDA-MB-231 cells. The transfection efficacy was confirmed by a Western blot analysis (**Figure 6E**). We then carried out a transwell assay under VWCE and WDR1-overexpression. The results show that the mean cell number in the MDA-MB-231-VWCE-WDR1 group that moved to the lower chamber was significantly increased compared to the MDA-MB-231-VWCE group. Similarly, in the MDA-MB453-VWCE-WDR1 group, the mean number of cells that moved to the lower chamber was significantly increased compared to the MDA-MB453-VWCE group (**Figure 6F**). The wound healing assay revealed that the relative mobility from 0 h to 24 h in the OE-WDR1 group was increased in the MDA-MB-231 and MDA-MB453 cells lines (**Figure 6G**). The wound healing assay showed that the overexpression of WDR1 partially reversed the suppressive role of VWCE on tumor migration in MDA-MB-231 and MDA-MB-453 breast cancer cell lines. In the transwell assay, WDR1 overexpression partially reversed the suppressor role of VWCE on tumor invasion in the MDA-MB-231 and MDA-MB-453 breast cancer cell lines. In the colony formation assay, there was an increased number of cell colonies in the OE-WDR1 group in the presence of TMZ (**Figure 6H**). Thus, WDR1 overexpression partially reversed the tumor proliferation suppressor role of VWCE in the MDA-MB-231 and MDA-MB-453 breast cancer cell lines.

### VWCE Expression Is Associated With Breast Cancer Clinicopathology and Survival

**Figure 7:**
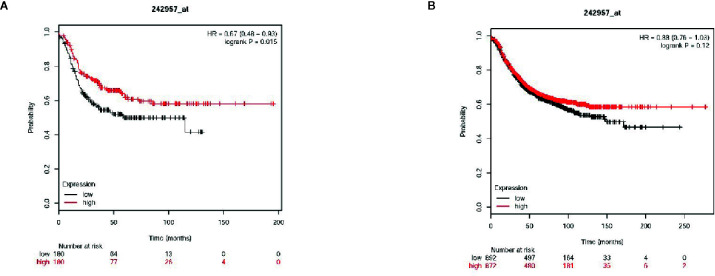
Von Willebrand factor C and EGF domain (VWCE) expression is associated with breast cancer clinicopathology and survival. Kaplan-Meier survival plots demonstrating the good prognostic effect of VWCE overexpression in breast cancer patients. **(A)** Overall survival (OS) rate for triple negative breast cancer patients with low VWCE expression (n=180) compares to those with high VWCE expression (n=180), **(B)** OS rate for breast cancer patients with low VWCE expression (n=497) compares to those with high VWCE expression (n=480).

Next, the association between the level of VWCE expression and clinical characteristics was further assessed. A tissue microarray (TMA) for both an immunohistochemical and statistical analysis of the clinicopathological data for the 87 breast cancer patients included in the array indicated that the level of VWCE expression was significantly correlated with the histological grade (p < 0.001) of the tumor, but not with the age of the patient or type of breast cancer (**Table 1**). Finally, according to the microarray data from 3,955 breast cancer patients, the results of the Kaplan-Meier Plotter analysis (www.kmplot.com) showed that a higher VWCE (242957_at) abundance correlated with a better overall survival (OS). A higher VWCE (242957_at) abundance was correlated with a better OS based on the microarray data from 360 triple negative breast cancer patients (**Figure 7A**). A higher VWCE (242957_at) abundance was also correlated with a better OS based on the microarray data from 970 breast cancer patients (**Figure 7B**). Based on these results, we concluded that the down-regulation of VWCE was associated with TNBC progression and may serve as a novel prognostic biomarker for TNBC.

**Table 1:** Association of VWCE protein expression and clinicopathologic characteistics.

Clinicopathologic variables	Number of cases	VWCE expresston		p
		High	Low	
All cases	87	34	53	
Gender				
Female	87	34	53	
Age				
≤60	40	11	29	0.333
>60	47	15	22	
Histopathological grade				
Low(Gl+G2)	61	13	48	0.001
High (G3+G4)	26	21	5	
Histotypes				
Invasive ductal carcinoma	74	29	45	1.000
Invasive lobular carcinoma	13	5	8	

*P < 0.05 by χ2 test.

## Discussion

Here, we found that normal breast tissue samples exhibited a higher level of VWCE expression than breast cancer tissues. For the first time, we have fully revealed the potential role of VWCE in breast cancer progression, and found that VWCE is associated with the inhibition of breast cancer metastasis (e.g., tumor cell growth, migration, invasion, and epithelial-interstitial transformation [EMT]). Moreover, VWCE inhibits the growth and migration of cancer cells by limiting the expression of WD-repeat domain 1 (WDR1). WDR1 is highly conserved across all eukaryotes, and has been shown to act together with ADF/cofilin to promote actin dynamics in various actin-based processes, including cell migration and cytokinesis (23–25). Thus, our findings suggest that VWCE may be a novel tumor suppressor that limits breast cancer progression by regulating WDR1 expression.

The findings of this study show that VWCE is significantly downregulated in breast cancer. Importantly, VWCE expression was significantly associated with the OS of breast cancer patients. Together, these findings indicate that VWCE may represent a useful prognostic biomarker for breast cancer. Recently, the upregulation of VWCE has been found in prostate cancer (26); however, its biological role in breast cancer remains unknown. In the current study, we have demonstrated for the first time, that VWCE is down-regulated in breast cancer and inhibits the proliferation, migration, invasion and metastasis of breast cancer cells both *in vitro* and *in vivo*. Breast cancers differ somewhat from other cancers. VWCE is also called Upregulated gene 11 (URG11), a gene upregulated by Heptatitis B Virus X protein (HBx) (27). URG11 mRNA and protein were observed in HB x Ag-positive compared to HB x Ag-negative HepG2 cells (14). Antibodies to URG11 were detectable in serum samples from patients with HBV associated cirrhosis and HCC, but not in serum samples from uninfected individuals (28), but the reason is unclear. It suggests that this gene may be related to inflammation. Hepatocellular carcinoma, gastric cancer and lung cancer are all cancers related to inflammation, while breast cancer is not very related to inflammation. This means that the role of VWCE in breast cancer is different from that of other inflammation-related tumors. This indicates that VWCE plays a vital role in breast cancer.

Abnormal WDR1 expression has been reported to be associated with breast cancer metastasis (29). Moreover, a WDR1 deletion was found to result in defective E-cadherin distribution (30, 31). In these studies, WDR1 was identified as an oncogene for the development of oncogenic breast cancer. In the present study, we demonstrated that VWCE down-regulates the expression of WDR1 using proteomic techniques. Importantly, the overexpression of WDR1 could effectively block the ability of VWCE to inhibit proliferation and metastasis. The findings of our study provide solid evidence that VWCE inhibits breast cancer proliferation and metastasis by targeting WDR1. We will further investigate the effect of VWCE on WDR1 in subsequent genetic studies.

The EMT process also plays an important role in both tumor cell metastasis and chemical resistance (32, 33). In addition, VWCE promotes tumor cell migration and invasion through EMT, as VWCE overexpression leads to decreased levels of N-cadherin, vimentin, and the EMT-induced transcription factor, ZEB1, as well as elevated E-cadherin levels, indicating a significant inhibition of EMT processes. WDR1 enhances the effect of MRTF-A-induced breast cancer cell migration by promoting the expression of EMT markers and migration markers by RhoA-MRTF (34). WDR1-mediated actin dynamics are required for membrane-associated adhesive junction remodeling in tumor cells. Based on the above results, VWCE appears to mediate WDR1-mediated disruption of actin dynamics, and cytoskeletal reorganization may be a target for tumor therapy intervention.

Notably, VWCE overexpression inhibited the proliferation and metastasis of mouse breast cancer cells, suggesting that VWCE is a potential therapeutic target for breast cancer. Interestingly, our mechanical and functional data allow us to better understand the functional role of WDR1 in human breast cancer. Moreover, its expression can positively regulate cellular proliferation, migration, invasion, and metastasis. In addition, WDR1, a direct functional target of VWCE in breast cancer, may represent an attractive therapeutic target since it can be precisely targeted with specific antibodies. The main hypothesis we can draw from this study is that an increased level of VWCE as a regulator of WDR1 may represent a therapeutic strategy for breast cancer; however, this attractive assumption requires further validation in animal models.

In conclusion, this study found that VWCE is a potent tumor suppressor in breast cancer, and its growth inhibition is mediated in part by the expression of its downstream target gene, WDR1. Using cell culture and animal models, the functional characterization of the role of VWCE revealed that the loss of its expression is an important event in the progression of breast cancer.

## Data Availability Statement

The original contributions presented in the study are publicly available. This data can be found here: The ProteomeXchange Consortium (http://proteomecentral.proteomexchange.org) with the dataset identifier PXD021733.

## Ethics Statement

The studies involving human participants were reviewed and approved by: Clinical research medical ethics committee of the First Affiliated Hospital of Shenzhen University. The patients/participants provided their written informed consent to participate in this study. The animal study was reviewed and approved by Clinical research medical ethics committee of the First Affiliated Hospital of Shenzhen University.

## Author Contributions

NX designed the study. DZ performed most of the experiments. LW performed part of the experiments. WL made some suggestions and help on the study. DZ and LL analyzed the data. DZ prepared and wrote manuscript. SH and FY guided the preliminary experiments and revised the manuscript. All authors contributed to the article and approved the submitted version.

## Funding

This project was supported by the Natural Science Foundation of National (81972003), the Natural Science Foundation of Guangdong (2016A030313029, 2017A030313668), the Sanming Project of Medicine in Shenzhen (SZSM201612031), and the Shenzhen Municipal Government of China (JCYJ20170817171808368, JCYJ20170818085657917, JCYJ20180507184647104, KQTD20170810160226082).

## Conflict of Interest

The authors declare that the research was conducted in the absence of any commercial or financial relationships that could be construed as a potential conflict of interest.

## References

[1] Bray F Ferlay J Soerjomataram I Siegel RL Torre LA Jemal A Global cancer statistics 2018: GLOBOCAN estimates of incidence and mortality worldwide for 36 cancers in 185 countries. CA Cancer J Clin (2018) 68:394–424. 10.3322/caac.21492 30207593

[2] Kennecke H Yerushalmi R Woods R Cheang MC Voduc D Speers CH Metastatic behavior of breast cancer subtypes. J Clin Oncol (2010) 28:3271–7. 10.1200/JCO.2009.25.9820 20498394

[3] Chaffer CL Weinberg RA A perspective on cancer cell metastasis. Science (2011) 331:1559–64. 10.1126/science.1203543 21436443

[4] Zhou YF Eng ET Zhu J Lu C Walz T Springer TA Sequence and structure relationships within von Willebrand factor. Blood (2012) 120:449–58. 10.1182/blood-2012-01-405134 PMC3398765 22490677

[5] Malukiewicz-Wisniewska G Kotschy M Von Willebrand factor in subretinal fluid. Eur J Ophthalmol (2001) 11:361–5. 10.1177/112067210101100408 11820308

[6] Szpera-Gozdziewicz A Majcherek M Boruczkowski M Gozdziewicz T Dworacki G Wicherek L Circulating endothelial cells, circulating endothelial progenitor cells, and von Willebrand factor in pregnancies complicated by hypertensive disorders. Am J Reprod Immunol (2017) 77. 10.1111/aji.12625 28224722

[7] Sheldon TJ Miguel-Aliaga I Gould AP Taylor WR Conklin D A novel family of single VWC-domain proteins in invertebrates. FEBS Lett (2007) 581:5268–74. 10.1016/j.febslet.2007.10.016 18028914

[8] Kirschbaum M Jenne CN Veldhuis ZJ Sjollema KA Lenting PJ Giepmans B Transient von Willebrand factor-mediated platelet influx stimulates liver regeneration after partial hepatectomy in mice. Liver Int (2017) 37:1731–7. 10.1111/liv.13386 28178387

[9] Fiebig JE Weidauer SE Qiu LY Bauer M Schmieder P Beerbaum M The clip-segment of the von Willebrand domain 1 of the BMP modulator protein Crossveinless 2 is preformed. MOLECULES (2013) 18:11658–82. 10.3390/molecules181011658 PMC6270503 24071977

[10] Zhang JL Huang Y Qiu LY Nickel J Sebald W von Willebrand factor type C domain-containing proteins regulate bone morphogenetic protein signaling through different recognition mechanisms. J Biol Chem (2007) 282:20002–14. 10.1074/jbc.M700456200 17483092

[11] Chapellier M Maguer-Satta V BMP2, a key to uncover luminal breast cancer origin linked to pollutant effects on epithelial stem cells niche. Mol Cell Oncol (2016) 3:e1026527. 10.1080/23723556.2015.1026527 27314065 PMC4909443

[12] Bosukonda A Carlson WD Harnessing the BMP signaling pathway to control the formation of cancer stem cells by effects on epithelial-to-mesenchymal transition. Biochem Soc Trans (2017) 45:223–8. 10.1042/BST20160177 28202676

[13] Fan R Li X Du W Zou X Du R Zhao L Adenoviral-mediated RNA interference targeting URG11 inhibits growth of human hepatocellular carcinoma. Int J Cancer (2011) 128:2980–93. 10.1002/ijc.25624 20725996

[14] Lian Z Liu J Li L Li X Tufan NL Clayton M Upregulated expression of a unique gene by hepatitis B x antigen promotes hepatocellular growth and tumorigenesis. Neopalsia (2003) 5:229–44. 10.1016/S1476-5586(03)80055-6 PMC1502406 12869306

[15] Tong GD Zhang X Zhou DQ Wei CS He JS Xiao CL Efficacy of early treatment on 52 patients with preneoplastic hepatitis B virus-associated hepatocellular carcinoma by compound Phyllanthus Urinaria L. Chin J Integr Med (2014) 20:263–71. 10.1007/s11655-013-1320-7 23529834

[16] Peng W Zhang J Liu J URG11 predicts poor prognosis of pancreatic cancer by enhancing epithelial-mesenchymal transition-driven invasion. Med Oncol (2014) 31:64. 10.1007/s12032-014-0064-y 24930007

[17] Du R Xia L Sun S Lian Z Zou X Gao J URG11 promotes gastric cancer growth and invasion by activation of beta-catenin signalling pathway. J Cell Mol Med (2010) 14:621–35. 10.1111/j.1582-4934.2008.00622.x PMC3823461 19413886

[18] Liu ZL Wu J Wang LX Yang JF Xiao GM Sun HP Knockdown of Upregulated Gene 11 (URG11) Inhibits Proliferation, Invasion, and beta-Catenin Expression in Non-Small Cell Lung Cancer Cells. Oncol Res (2016) 24:197–204. 10.3727/096504016X14648701447850 27458101 PMC7838721

[19] Florell SR Coffin CM Holden JA Zimmermann JW Gerwels JW Summers BK Preservation of RNA for functional genomic studies: a multidisciplinary tumor bank protocol. Mod Pathol (2001) 14:116–28. 10.1038/modpathol.3880267 11235903

[20] Sharma C Wang HX Li Q Knoblich K Reisenbichler ES Richardson AL Protein Acyltransferase DHHC3 Regulates Breast Tumor Growth, Oxidative Stress, and Senescence. Cancer Res (2017) 77:6880–90. 10.1158/0008-5472.CAN-17-1536 PMC5819883 29055014

[21] Zhang F Zhang H Wang Z Yu M Tian R Ji W P-glycoprotein associates with Anxa2 and promotes invasion in multidrug resistant breast cancer cells. Biochem Pharmacol (2014) 87:292–302. 10.1016/j.bcp.2013.11.003 24239898

[22] Yan M Wang J Ren Y Li L He W Zhang Y Over-expression of FSIP1 promotes breast cancer progression and confers resistance to docetaxel via MRP1 stabilization. Cell Death Dis (2019) 10:204. 10.1038/s41419-018-1248-8 30814489 PMC6393503

[23] Bamburg JR Proteins of the ADF/cofilin family: essential regulators of actin dynamics. Annu Rev Cell Dev Biol (1999) 15:185–230. 10.1146/annurev.cellbio.15.1.185 10611961

[24] Ono S Regulation of actin filament dynamics by actin depolymerizing factor/cofilin and actin-interacting protein 1: new blades for twisted filaments. Biochemistry-US (2003) 42:13363–70. 10.1021/bi034600x 14621980

[25] Okada K Blanchoin L Abe H Chen H Pollard TD Bamburg JR Xenopus actin-interacting protein 1 (XAip1) enhances cofilin fragmentation of filaments by capping filament ends. J Biol Chem (2002) 277:43011–6. 10.1074/jbc.M203111200 12055192

[26] Sun C Zhang G Cheng S Qian H Li D Liu M URG11 promotes proliferation and induced apoptosis of LNCaP cells. Int J Mol Med (2019) 43:2075–85. 10.3892/ijmm.2019.4121 PMC6443344 30864678

[27] Lian Z Liu J Li L Li X Clayton M Wu MC Enhanced cell survival of Hep3B cells by the hepatitis B x antigen effector, URG11, is associated with upregulation of beta-catenin. Hepatology (2006) 43:415–24. 10.1002/hep.21053 16496348

[28] Tong GD Zhou DQ He JS Xiao CL Liu XL Zhou XZ Preneoplastic markers of hepatitis B virus-associated hepatocellular carcinoma and their significance in clinical settings. Zhonghua Gan Zang Bing Za Zhi (2007) 15:828–32. 18073065

[29] Lee JH Kim JE Kim BG Han HH Kang S Cho NH STAT3-induced WDR1 overexpression promotes breast cancer cell migration. Cell Signal (2016) 28:1753–60. 10.1016/j.cellsig.2016.08.006 27521604

[30] Xu J Wan P Wang M Zhang J Gao X Hu B AIP1-mediated actin disassembly is required for postnatal germ cell migration and spermatogonial stem cell niche establishment. Cell Death Dis (2015) 6:e1818. 10.1038/cddis.2015.182 26181199 PMC4650729

[31] Chu D Pan H Wan P Wu J Luo J Zhu H AIP1 acts with cofilin to control actin dynamics during epithelial morphogenesis. Development (2012) 139:3561–71. 10.1242/dev.079491 22899846

[32] Morris HT Machesky LM Actin cytoskeletal control during epithelial to mesenchymal transition: focus on the pancreas and intestinal tract. Br J Cancer (2015) 112:613–20. 10.1038/bjc.2014.658 PMC4333498 25611303

[33] Fischer KR Durrans A Lee S Sheng J Li F Wong ST Epithelial-to-mesenchymal transition is not required for lung metastasis but contributes to chemoresistance. NATURE (2015) 527:472–6. 10.1038/nature15748 PMC4662610 26560033

[34] Xiang Y Liao XH Yao A Qin H Fan LJ Li JP MRTF-A-miR-206-WDR1 form feedback loop to regulate breast cancer cell migration. Exp Cell Res (2017) 359:394–404. 10.1016/j.yexcr.2017.08.023 28822708

